# COVID-19 Vaccine hesitancy and influencing factors: An example from Turkey

**DOI:** 10.12669/pjms.40.4.7979

**Published:** 2024

**Authors:** Didem Sarimehmet, Yakup Kadri Sarimehmet, Bahar Candas Altinbas, Cuneyt Ardic

**Affiliations:** 1 Didem Sarimehmet, Lecturer, Vocational School of Health Services, Department of Medical Services and Techniques, Karadeniz Technical University, Trabzon, Turkey; 2 Yakup Kadri Sarimehmet, Research Asisstant, Faculty of Medicine, Department of Family Medicine. Recep Tayyip Erdogan University, Rize, Turkey; 3 Bahar Candas Altinbas, Assistant Professor, Faculty of Health Sciences, Department of Nursing. Karadeniz Technical University, Trabzon, Turkey; 4 Cüneyt Ardiç, Associate Professor, Faculty of Medicine, Department of Family Medicine. Recep Tayyip Erdogan University, Rize, Turkey

**Keywords:** COVID-19, Hesitancy, Pandemics, Vaccination

## Abstract

**Objective:**

To find out the opinions concerning vaccine hesitancy of people and influencing factors who had not received COVID-19 vaccination.

**Methods:**

This is a descriptive cross-sectional study. It was carried out between February and April 2022 with individuals who were not vaccinated against COVID-19. It included 634 participants registered at a family health center in Rize, Turkey. Data were collected by telephone using a questionnaire. For statistical analysis, the R programming language was used. The Boruta algorithm was used to rank the variables associated to the reasons for not trusting the vaccine.

**Results:**

“I do not trust vaccines (67%)” is the most frequently cited reason for not being vaccinated. The most often cited reasons for not trusting vaccinations are that vaccines are produced for the benefit of foreign companies (56.2%), vaccines are ineffective (55.5%), and vaccines have not undergone sufficient scrutiny (53.2%). According to Boruta analyses, the top three variables most closely associated with not trusting COVID-19 vaccines were belief that vaccines are produced for the benefit of foreign countries/vaccines companies, imported vaccines have not undergone sufficient scrutiny, and vaccines being ineffective.

**Conclusions:**

People do not get vaccinated because they do not trust vaccinations due to concerns about their safety, effectiveness, political influences, and potential adverse effects.

## INTRODUCTION

Vaccination is the most comprehensive, effective, and safe public health measure in the fight against the epidemic.^1^ Vaccines are one of the most important achievements of the century and contribute greatly to the decline in morbidity and mortality from infectious diseases.^2^ Some 13 billion doses of the COVID-19 vaccine had been administered by October 19, 2022.^3^ In the data for Turkey show that approximately 58 million first doses were administered and 53 million second doses, the total number of COVID-19 vaccines administered being approximately 150 million.^4^

Although vaccination is a successful means of protecting and improving health, questioning their safety and the need for vaccines has led to the concept of vaccine hesitancy.^5^ This refers to an individual’s hesitation over receiving the vaccine, or reluctance or refusal to be vaccinated when vaccination services are available in a community.^2^,^6^ The WHO cited vaccine hesitancy as one of the 10 principal threats to global health in 2019.^7^,^8^ Numerous factors affect vaccine hesitancy, such as communications, the media, historical influences, religion, culture, gender, socioeconomic level, politics, geographical barriers, experience of vaccination, and vaccination program design.^1^ De Figueiredo et al. (2020) examined vaccine hesitancy in 149 countries and reported higher hesitancy rates in Europe than in other continents.^9^

Vaccines manufactured against COVID-19 and the need for vaccination have fueled debates about vaccine hesitancy.^1^,^5^ The principal causes of vaccine hesitancy and rejection of the COVID-19 vaccine include young age, loss of income during the pandemic, lack of confidence in the COVID-19 vaccine, perception of the information provided as inconsistent and contradictory, and experience of interruptions to healthcare during the pandemic. Soares et al.’s study of vaccine hesitancy toward COVID-19 in Portugal reported hesitancy among 56% of participants, with 9% refusing to be vaccinated.^1^

A study assessing public attitudes toward vaccination in Turkey found that 41.2% of participants wished to be vaccinated, while 37.9% were undecided. This study investigated reasons regarding vaccine hesitancy among individuals registered at a family health center in Turkey.^10^

## METHODS

This descriptive study was designed based on the STROBE checklist for reporting observational studies. The study population consisted of 1664 individuals registered at a family health center in Rize, Turkey, who were not vaccinated at the time of data collection (February 01, 2022). It was aimed to contact the entire population with no sample selection being performed. Inclusion criteria were age 18 years or more, absence of any mental health problems or hearing impairment, no general refusal of vaccination, and consent to participate in the study.

### Inclusion and Exclusion Criteria

The phone numbers of 315 individuals were found to be invalid/wrong, 223 individuals were generally opposed to all vaccination, 184 could not be reached despite three attempts to do so, 116 refused to participate in the study, 86 individuals had since been vaccinated, 64 were under 18 years of age, and 42 were deaf/hard of hearing, and these were all excluded from the analysis. The study was finally completed with 634 participants.

The data were collected using a questionnaire designed by the researchers in the light of the previous studies.^1^,^2^,^8^ Verbal informed consent was obtained from all those who volunteered to participate and were enrolled in the study.

The questionnaire used in the study was administered to the participants by phone. The researcher who is working as a family physician at the Rize Kalkandere Family Health called those participant. If individuals could not be reached on the first occasion, were not available, or wished to speak at a later time, a second and, depending on the situation, third call were made. If the participant still could not be reached after three calls, no further calls were made. Individuals who indicated that they were still not available after three calls were also not contacted again. The average time for phone data collection was 20 minutes, the duration varying depending on the participant’s age, hearing impairment status, education level, etc.

### Statistical Analysis

The statistical analysis and evaluation of significant variables based on their level of importance were conducted using the R programming language. Analysis results were expressed as frequencies (percentages). Significance was set at p<0.050.

We wanted to determine the variables related to the reasons for not trusting the vaccine according to the degree of importance. The Boruta algorithm uses an iterative approach to identify and eliminate variables that are deemed statistically insignificant. The Boruta algorithm is founded on the concept of mitigating the misleading impact of chance fluctuations and correlations by introducing random variables into the system and gathering outcomes from a set of random samples.

### Ethical approval

It was obtained from the university Ethics Committee under the approved number 2022/18 on January 20, 2022.

## RESULTS

The mean age of the participants was 34.89±12.50 years, 58.7% (n=372) were women, 56.8% (n=360) were married, 39.4% (n=250) worked in the private sector, and 39.6% (n=251) had a college degree. Additionally, 82.8% (n=525) had no medical personnel in their families, 52.4% (n=332) did not smoke, and 72.4% (n=459) did not consume alcohol. Twenty-one percent reported comorbidities, 72.9% (n=97) hypertension, 34.6% (n=46) diabetes, 28.6% (n=38) asthma, 18% (n=24) chronic heart failure, and 4.5% (n=6) chronic obstructive pulmonary disease.

The three most frequently cited reasons for not being vaccinated were “I do not trust vaccines” (67%), “I think having COVID-19 is sufficient to gain immunity” and “I think I can be protected against COVID-19 by adhering to hygiene methods or by supplements that boost immunity (50%) . Addionatally, reasons for not trusting to vaccines can be found in Table-I.

**Table-I:** Sociodemographic features (n=634)

Feature		n(%)
Age (Variable_1)		34.89±12.50 (18-75)^*^
Gender (Variable_2	Female	262 (41.3)
	Male	372 (58.7)
Marital status (Variable_3)	Married	360 (56.8)
	Single	274 (43.2)
Employment status (Variable_4)	Private sector	250 (39.4)
	Unemployed	244 (38.5)
	Clerical	86 (13.6)
	Retired	54 (8.5)
Education status (Variable_5)	Illiterate	12 (1.9)
	Literate	24 (3.8)
	Elementary school	175 (27.6)
	High school	251 (39.6)
	University or higher	172 (27.1)
Medical personnel in the family (Variable_6)	Yes	109 (17.2)
	No	525 (82.8)
Smoking (Variable_7)	Yes	302 (47.6)
	No	332 (52.4)
Alcohol use (Variable_8)	Yes	175 (27.6)
	No	459 (72.4)
Comorbidity (Variable_9)	Yes	133 (21)
	No	501 (79)

### Feature selection

**Fig.1:**
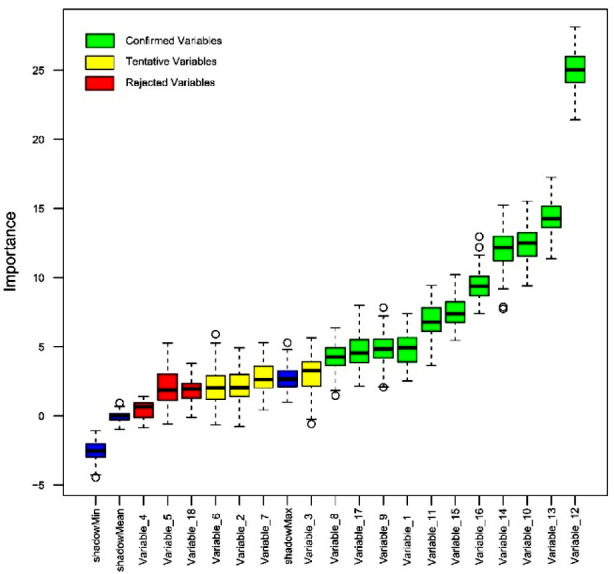
Variable importance.

The result of feature screening based on the Boruta algorithm is shown in Figure. 1. In order of Z-values, the 11 variables most closely associated with not trusting to COVID-19 vaccines were beliefs that vaccines are produced for the benefit of foreign countries/vaccines companies, imported vaccines have not undergone sufficient scrutiny, vaccines being ineffective, number of vaccines being administered is excessive, the vaccines being a political plan, concerns about serious side-effects, belief that the ingredients in the vaccines are harmful to health, age, comorbidities, belief that there is no COVID-19 disease, and alcohol use.Fig.1

A statistically significant association was found between not having trust in COVID-19 vaccines and having contracted COVID-19 (p=0.001), having a relative who had contracted COVID-19 (p=0.031), and losing a relative due to COVID-19 (p=0.022). Of those who do not trust the COVID-19 vaccines, 60.9% had contracted COVID-19, 81.6% have a relative who contracted COVID-19, and 32.2% have had a family member die due to COVID-19 (Table-II).

**Table-II:** Reasons for not being vaccinated and trusting to COVID-19 vaccines.

Reasons for not being vaccinated (n=634)	Yes n(%)	No n(%)	No idea n(%)
I do not trust vaccines	425 (67)	115 (18.1)	94 (14.8)
I think having COVID-19 is sufficient to gain immunity	317 (50)	179 (28.2)	175 (27.6)
I think I can be protected against COVID-19 by adhering to hygiene methods boost immunity (vitamins, fruit, etc.)	317 (50)	118 (18.6)	199 (31.4)
Even if I contract the disease, I think I can fight COVID-19 off because I am young	280 (44.2)	179 (28.2)	175 (27.6)
I trust the statements made by anti-vaccines doctors	139 (21.9)	271 (42.7)	224 (35.3)
I think the negative statements by eminent members of society are correct	130 (20.5)	287 (45.3)	217 (34.2)
I do not believe in the existence of COVID-19 disease	118 (18.6)	396 (62.5)	120 (18.9)
Reasons for not trusting to COVID-19 vaccines (n=425)			
I think the vaccines are ineffective (Variable_10)	236 (55.5)	64 (15.1)	125 (29.4)
I believe the ingredients in the vaccines are harmful to health (Variable_11)	203 (47.8)	115 (27.1)	107 (25.2)
I think the vaccines are produced for the benefit of foreign countries/vaccines companies (Variable_12)	239 (56.2)	80 (18.8)	106 (24.9)
I think imported vaccines have not undergone sufficient scrutiny (Variable_13)	226 (53.2)	98 (23.1)	101 (23.8)
I think the number of vaccines being administered is excessive (Variable_14)	200 (47.1)	77 (18.1)	148 (34.8)
I think the vaccines have serious side-effects (Variable_15)	184 (43.3)	142 (33.4)	99 (23.3)
I think the vaccines are a political plan (Variable_16)	193 (45.4)	132 (31.1)	100 (23.5)
I do not believe in the existence of COVID-19 disease (Variable 17)	74 (17.4)	257 (60.5)	94 (22.1)
I think that chips are being implanted through the vaccines (Variable_18)	52 (12.2)	330 (77.6)	43 (10.1)

**Table-III:** Relationships between reasons for not being trust COVID-19 vaccines and demographic data concerning COVID-19 (n=425)

	I do not trust vaccines				Statistical analysis

	Yes n(%)	No ides n(%)	No n(%)		
Having contracted COVID-19	Yes	259 (60.9)^a^	46 (48.9)^a,b^	50 (43.5)^b^	X^2^=13.432
	No	166 (39.1)^a^	48 (51.2)^a,b^	65 (56.5)^b^	P= 0.001
Having a relative who contracted COVID-19	Yes	347 (81.6)^a^	70 (74.5)^a,b^	82 (71.3)^b^	X^2^=6.960
	No	78 (18.4)^a^	24 (25.5)^a,b^	33 (28.7)^b^	P= 0.031
Death due to COVID-19	Yes	137 (32.2)^a^	26 (27.7)^a,b^	22 (19.1)^b^	X^2^=7.645
	No	288 (67.8)^a^	68 (72.3)^a,b^	93 (80.9)^b^	P= 0.022

Analysis revealed that 95.1% (n=603) of participants reported hearing about the vaccines from various sources. The three principal sources of that information were television (79.6%), the internet (64.3%), and social media (56.9%). Other sources of information are social environment (55.1%), newspaper (35.2%), physician (33.8%) and nurse (28.4 %).

## DISCUSSION

COVID-19 vaccines have been developed and approved for use in record time, and while many individuals have eagerly received the vaccine, there remain a significant number of individuals who are hesitant or outright refuse to get vaccinated. It could be argued that trust plays an inherent and adjustable role in the effective adoption of a COVID-19 vaccine.^11^ According to a study by Roy et al. one of the most frequently cited reason for not getting vaccinated is a lack of trust in vaccines.^12^ Specifically, 67% of respondents in the current study reported not trusting vaccines as a reason for not getting vaccinated. It is known that trusting in COVID-19 vaccines increases the willingness to accept the vaccines and booster doses.^13^ The current finding is consistent with previous research that has highlighted the role of vaccine hesitancy and mistrust in vaccine efficacy and safety.

Another commonly cited reason for vaccine hesitancy is the belief that prior COVID-19 infection can provide sufficient immunity and that adherence to hygiene practices can boost one’s immune system to protect against the disease. In a recent study, survey respondents indicated their strong agreement (24.3%) that natural immunity provides longer-lasting protection than vaccination, as well as agreement (38.8%) and strong agreement (21.1%) that natural exposure to germs and viruses affords the safest protection, with an additional 40.2% expressing agreement.^14^ Besides, İkiışık et al. reported that individuals believe that they can protect themselves against COVID-19 by natural and traditional means.^8^

The maintenance of clean water sources, proper sanitation practices, and adequate hygiene measures are critical components in safeguarding public health amidst COVID-19 pandemic.^15^ Consistent implementation of appropriate washing and waste disposal protocols can serve as additional preventive measures against person-to-person transmission of the COVID-19 pathogen. While it is true that prior infection does confer some level of immunity, current evidence suggests that the protection provided by vaccination is stronger and longer-lasting than that provided by natural infection alone.

A recent publication by Hacisuleyman et al. revealed that despite having a substantial level of antibodies, two previously infected individuals contracted new COVID-19 variants following vaccination.^16^ Therefore, a combined approach of vaccination and strict adherence to hygiene guidelines, may serve as a more effective strategy in curtailing the spread of COVID-19.

The study reveals that two noteworthy factors contributing to vaccine hesitancy are distrust in vaccines as an instrument of foreign countries or vaccine manufacturers, and the perception that vaccines are part of a political agenda. These findings are complementary, as they strengthen each other in highlighting the issue of vaccine hesitancy. The former reason indicates that a lack of trust in vaccines stems from the perception that they are produced for reasons that are not in the best interest of the recipient country or individuals. The latter reason suggests that vaccines are viewed as a tool that serves political interests, rather than being grounded in scientific evidence or public health goals. Together, these reasons reflect a complex interplay of political, social, and economic factors that underlie vaccine hesitancy. This concern is often rooted in a lack of trust in government and the pharmaceutical industry.^11^

A study by Peterson and colleagues (2022) found that mistrust of government and institutions significantly predicted vaccine hesitancy.^17^ However, it is important to note that the COVID-19 vaccines were developed by scientists and healthcare professionals, and their approval and distribution were based on rigor. Other reasons cited for vaccine hesitancy is the belief that imported vaccines have not undergone sufficient scrutiny, fear of side-effect, belief that the ingredients in the vaccines are harmful to health and concerns about vaccine effectiveness. Similarly, in different studies, lack of information about vaccines, unreliability, and fear of side effects have been identified as causes of vaccine hesitancy.^18^,^19^ These concerns are often related to the speed of vaccine development and approval. While it is understandable that some individuals may have concerns or reservations about the COVID-19 vaccine, it is important to address these concerns with accurate information and to promote vaccine acceptance.

However, studies have shown that the COVID-19 vaccines are highly effective in preventing COVID-19 and its severe outcomes. The safety and efficacy of COVID-19 vaccines have been rigorously tested and confirmed in large clinical trials, and numerous public health organizations have recommended their use.^20^,^21^ For example, a study by Polack and colleagues found that the Pfizer-BioNTech vaccine was 95% effective in preventing COVID-1 9.^22^ However, it is known that vaccines are widely recognized as a highly effective and optimal strategy for mitigating the substantial disease burden experienced around the world. They provide protection against disease by preventing infection, decreasing disease severity and mortality rates, and limiting the impact of pandemics on public health systems and national economies.^23^

As such, efforts should be made to educate individuals about the benefits of vaccination and to address misconceptions and misinformation about the vaccine. This can help to increase vaccine uptake and promote public health during the ongoing. The belief that the number of vaccines being administered is excessive is also cited as a reason for vaccine hesitancy. This concern may be related to misinformation and conspiracy theories that suggest vaccines are being used for population control or to harm individuals. However, there is no evidence to support these claims, and vaccines have been shown to be safe and effective in preventing infectious diseases.^20^

Most participants learned about the vaccines through mass media such as television, the internet, and social media. Mass media play an important role in informing and educating society. 24,25 However, incorrect information from the uncontrolled use of mass media can influence individuals’ attitudes and perceptions toward the vaccine.^26^ Any incomplete or incorrect information about the vaccine may pave the way for the adoption of anti-vaccination methods. Furthermore, the spread of misinformation can result in a reluctance to share accurate information, thus further complicating public health efforts. In Yıldız et al.’s study, social media ranked third (18%) among the reasons for vaccination hesitancy.^27^

However, studies have found that only 5.86% of tweets shared on social media concerning vaccines were based on scientific sources.^28^ Surprisingly, in the present study, health professionals represented the least common source of information about vaccines. It may therefore be concluded that inaccurate and unreliable information in the mass media spreads rapidly and had undesirable effects on the decision-making processes of individuals who already exhibited vaccine hesitancy during the pandemic. Society should therefore be encouraged to obtain information about vaccination from scientific sources and health professionals.

### Limitations

The study was carried out with individuals in a province in the eastern Black Sea region. However, thoughts on vaccine hesitancy may show cultural or regional differences. Therefore, the results of the study cannot be generalized in other provinces.

## CONCLUSION

The main barriers to overcoming vaccine hesitancy in COVID-19 are concerns about safety, efficacy, and possible adverse side-effects, a finding consistent with studies of different populations in various countries. The mass media were the most common source of information about COVID-19 and vaccines. Our participants believed that they could protect themselves against COVID-19 by adhering to hygiene measures or taking immunity-boosting supplements. Most participants trusted Turkish scientists and would consider receiving the Turkish vaccine after seeing the results, but not immediately.

### Authors Contribution:

**DS:** Conceived and designed the study, edited the manuscript, and is responsible for the integrity of the research.

**YKS:** Designed the study, conducted data collection, and contributed the manuscript.

**BCA:** Conducted data collection and performed statistical analysis.

**CA:** Reviewed and provided final approval for the manuscript.
